# The value of biomarkers in the therapy of CRSwNP with biologicals—a long-term follow-up of dupilumab therapy

**DOI:** 10.1007/s00405-024-08574-4

**Published:** 2024-05-06

**Authors:** Sven Ole Sarnoch, Amra Pepić, Lisa Schmitz, Benjamin Becker, Christian Betz, Anna-Sophie Hoffmann

**Affiliations:** 1 Department of Otorhinolaryngology, University Medical Center Hamburg-Eppendorf, Martinistraße 52, 20246 Hamburg, Germany; 2 Institute of Medical Biometry and Epidemiology, University Medical Center Hamburg-Eppendorf, Martinistraße 52, 20246 Hamburg, Germany

**Keywords:** Dupilumab, CRSwNP, Serum IgE, Type 2 inflammation, Real-world-data

## Abstract

**Purpose:**

Since its release, Dupilumab has shown great results in treating severe uncontrolled CRSwNP. However, there is a lack of real-world data beyond 12 months of follow-up, and it is not clear to what extent biomarkers are appropriate for monitoring and predicting the Dupilumab therapy success. Hence, this study aims to analyze biomarkers for monitoring therapy, predicting therapy success and assess the effect of Dupilumab in real-world settings.

**Methods:**

The follow-up was performed with 104 patients retrospectively up to 22 months, assessing SNOT-22, NPS, olfactometry, ACS, FEV-1, and blood biomarkers (total serum IgE, Eosinophils, ECP). Patients were divided into subgroups depending on their pretherapeutic biomarker levels and subsequent development was analyzed.

**Results:**

There was substantially improvement in all clinical parameters up to 1 year and then continuously up to month 22. Patients with initially elevated baseline blood eosinophil counts (> 0.5 billion/L) had a trend of better SNOT-22 development after 1 year (− 12.19 points, *p* = 0.03). The course of total serum IgE showed moderate correlation with almost all clinical variables obtained. Therapy was well tolerated with only mild and transient adverse events.

**Conclusion:**

Dupilumab has considerably reduced symptoms and disease severity even beyond 1 year of treatment, supporting its role as targeted and effective treatment option for CRSwNP. Our data shows that total serum IgE is a promising biomarker for the monitoring during the treatment with Dupilumab. Elevated pre-therapeutic serum eosinophil counts may be a predictor of good subjective response to therapy. Larger cohorts and a long-term-follow-up over years are needed to further consolidate these findings.

## Introduction

With a prevalence of 5–12%, chronic rhinosinusitis (CRS) is one of the most common chronic diseases worldwide [1, 2]. A severe subtype of CRS is chronic rhinosinusitis with nasal polyps (CRSwNP), characterized by bilateral inflammation of the nose, nasal cavities and inflammatory polypoid outgrowths from the mucosa [2]. CRSwNP accounts for approximately 25% to 30% of all CRS cases and with an overall prevalence between 2.11 and 4% in Europe, CRSwNP contributes to the majority of CRS treatment costs, presenting enormous socioeconomic and therapeutic challenges to healthcare systems [1, 3]. Its major clinical symptoms reach from nasal obstruction, loss of olfactory function, anterior and posterior rhinorrhea to facial pressure pain and sleep disorder, leading to significant decline in health-related quality of life [4]. The symptom burden has been reported to be comparable with other chronic diseases such as diabetes or chronic obstructive pulmonary disease (COPD), and surprisingly, often found to be worse than with Parkinson’s disease or chronic heart failure [5–7].

### Type 2 inflammation

CRSwNP is classified as a type 2 inflammation-driven disease and frequently associated with comorbidities such as nonsteroidal anti-inflammatory drug exacerbated respiratory disease (N-ERD), asthma, allergies, as well as higher recurrence rates of nasal polyps after sinus surgery [8]. The hallmark of CRSwNP pathophysiology is the loss of healthy barrier function in sinonasal epithelial cells due to decreased epithelial resistance and increased permeability [9]. This type 2 inflammation process is characterized by eosinophil, lymphocyte, basophil and mast cell infiltration into polyp tissue, driven by T helper 2 (Th2) lymphocytes and group 2 innate lymphoid cells (ILC2) that produce high levels of interleukin-4 (IL-4), IL-5 and IL-13, which are considered key drivers of type 2 inflammation [9–11]. IL-13 plays a crucial role causing mucus hypersecretion, smooth muscle modification, fibrosis, increased airway activity and hyperreactivity [10]. IL-4 initiates downstream signaling pathways that result in a class switch of B-cell immunoglobulins in favor of IgE and IgG4, as well as the upregulation of IgE receptors on the surfaces of B-lymphocytes, mast cells and basophils [10, 12]. Both IL-4 and IL-13 are capable of producing eosinophil promoting factors such as IL-5 and eotaxins, which can stimulate effector cells like eosinophils to differentiate in bone marrow and migrate from blood to sites of inflammation [13]. Le Floc et al. further demonstrated the prominent role of both cytokines in the pathogenesis of type 2 inflammatory diseases, showing their capability of upregulating IgE receptors on mast cells independently of the presence of IgE, priming them for allergen-induced activation [10].

### Treating CRSwNP

While the foundation of treating CRSwNP was anti-inflammatory treatment with intranasal corticosteroids, short-term oral corticosteroids, and functional endoscopic sinus surgery (FESS), disease control was often incomplete and up to 40% were found to remain symptomatic or experience recurrences [14–16]. In recent years, the type 2 inflammation key cytokines IL-4 and IL-13 have become target of CRSwNP therapy by specifically blocking their inflammatory signaling pathway with anti-IL-4Rα monoclonal antibody Dupilumab (Dupixent®, Sanofi, Paris, France and Regeneron, NY, US) [17]. In the pivotal studies LIBERTY NP SINUS-24 (NCT02912468) and LIBERTY NP SINUS-52 (NCT02898454). Dupilumab demonstrated substantial disease control, better health related quality of life and symptom improvement versus placebo, leading to approval for treatment of CRSwNP in addition to atopic dermatitis and bronchial asthma [18, 19]. While these studies provide evidence of efficacy, real-world data is urgently needed to evaluate practical therapeutic effectiveness and to bridge the gap between controlled trials and daily patient care [20]. Long-term follow-up of Dupilumab treatment beyond 12 months is lacking so far.

### Biomarkers in biologics therapy

While there has been increased understanding of the pathology and signaling involved in type 2 inflammation in CRSwNP, the analysis in biomarkers influenced by biologics therapy is largely unrecorded. Furthermore, the availability of biomarkers which may serve as predictors of therapy response to biologics is limited. This is due to the absence of clinical trials validating laboratory findings, as well as the absence of standardized criteria for measuring biomarkers and assessing outcomes [21]. Considering the substantial financial burden associated with treatment costs and a certain rate of non-responders, it is crucial to predict treatment response and to evaluate whether certain patient groups are particularly suitable for targeted Dupilumab therapy before initial administration [21]. However, despite CRSwNP being characterized as type 2 inflammation disease, it remains uncertain if type 2 biomarkers like blood eosinophil counts (BECs), eosinophilic cationic protein (ECP) or serum IgE can predict sensitivity to Dupilumab and allow conclusions to be drawn about treatment success [22].

The ideal biomarker for clinical practice should be easy and minimally invasive to collect, such as through blood samples or nasal secretion fluid. It should be quantifiable, reproducible, and best directly involved in the pathogenesis of CRSwNP [21]. Currently available data suggest no, or at best, weak correlation between biomarkers and clinically detectable outcome parameters [18]. Nevertheless, exploring the connections between biomarkers and treatment response by analyzing the correlation between changes in biomarker levels and clinical parameters over time of treatment could provide insight into impact of biomarkers in disease processes, mechanisms of treatment and identify possible biomarker-based therapy monitoring in real-life settings [18, 21].

This study provides follow-up data for up to 22 months of Dupilumab treatment in CRSwNP, examining clinical and laboratory parameters. A primary focus was to investigate easy to obtain type-2 biomarkers like total serum IgE, BECs and ECP from peripheral blood to determine whether they are suitable for monitoring the treatment in routine clinical practice and to evaluate if they might also be suitable for predicting therapy success before first administration of Dupilumab.

## Materials and methods

This single-arm, longitudinal, single center cohort study assesses the impact of Dupilumab therapy on all patients with CRSwNP treated with Dupilumab at University Medical Center Hamburg-Eppendorf in Germany from November 2019 to May 2023. Informed consent was obtained from each participant. The study was approved by the Ethics Committee of the University of Hamburg (2020-10264-BO-ff).

All patients diagnosed with severe, uncontrolled CRSwNP according to EPOS criteria with subsequent guideline-based prescription of Dupilumab 300 mg s.c. every 2 weeks were included [2]. The EPOS guidelines define CRSwNP as uncontrolled when typical therapy and surgical therapy do not provide sufficient symptom relief. Additionally, patients must have met the following criteria to receive Dupilumab: significant loss of olfaction, significant reduction in quality of life (SNOT-22 score > 40 points), presence of bronchial asthma, or evidence of type-2 inflammation (≥ 10 eosinophils per high-power field in tissue samples or ≥ 100 kU/L of total serum IgE or ≥ 250 eosinophils per μL in blood samples). The need for systemic glucocorticoids at least twice a year was considered but not mandatory. Patients with cystic fibrosis, immunosuppressive diseases, or those receiving treatment with other biologics were excluded from the study, as well as pregnant and breastfeeding participants.

The baseline assessment of outcome parameters was established before the first medically monitored administration of Dupilumab. Subsequentially, follow-up visits were held with a fixed schedule after 4 weeks and then every 12 weeks at month 4, 7, 10 and 13 after the baseline visit. Thereafter, depending on the success of the therapy, treatment was either continued in the outpatient setting or followed by semi-annually check-ups. All patients who had at least one follow-up visit at month 4 were included in the analysis. Data was collected up to a maximum of 22 months.

### Study outcome parameters

Demographic data, body mass index (BMI), the Lund-Mackay computed tomography score (LMK), allergy status, presence of bronchial asthma, N-ERD status using the diagnostic criteria of the EACCI position paper [8], and number of previous sinusitis surgeries were recorded during the baseline visit. The baseline and each subsequent visit included the collection of blood parameters such as ECP (µg/L), absolute BECs (billion/L) and total serum IgE (IU/mL). At each visit, pulmonary function diagnostics was performed for evaluating forced expiratory volume in 1 s value (FEV-1). General and subjective sinusitis symptom severity such as nasal obstruction, rhinorrhea, facial pain, and sleep disorder were determined using the Visual Analog Scale (VAS). Olfactory function was screened using Sniffin-Sticks 12-identification test (Burghart Messtechnik GmbH) with the best performing results included. The NPS of each nasal cavity was determined according to the classification system of Gaevert [9, 10]. The Asthma Control Test (ACT) [11] and Sino-Nasal-Outcome Test-22 (SNOT-22) [12] were used.

NPS, VAS-scores and SNOT-22 were set as primary endpoints. Biomarkers (serum IgE, BECs, ECP), FEV-1, ACT and olfactometry performance were set as secondary endpoints.

### Statistics

The Statistical analyses were conducted using the statistical software R version 4.2.2 [23]. Missing data was not imputed. Descriptive statistics included mean and standard deviation (SD) for continuous variables, and frequencies with percentages for categorical variables.

To capture linear trends, mixed linear models were used, treating time as the fixed effect and patient ID as the random effect. The target variable was the respective change from baseline, with the baseline variable as a covariate. Results encompass adjusted mean changes from baseline with 95% confidence intervals (CI) for each time point.

Correlation analyses utilized Pearson correlation coefficient. Considering clustered data (multiple measurements per patient), correlations were calculated accounting for within-patient variability. First, random intercept models were employed to capture within cluster variability as random effects. Then, the conditional mean of these effects was subtracted from empirical absolute outcome values. Pearson correlation coefficient was computed for each comparison. All analyses are performed in an explorative manner. No adjustment for multiple testing was carried out.

The patients were stratified into two groups based on their baseline biomarker levels to assess potential differential progression with respect to the outcome parameters during the course of therapy (threshold values: total serum IgE 100 IU/mL, BECs 0.5 and 0.3 billion/L, ECP 30 µg/L).

## Results

A total of 104 patients (53 female, 51 male) treated with Dupilumab for CRSwNP were included in the study (Table 1). The mean age was 50.3 years (SD = 13.8), with a minimum age of 14 and maximum age of 84. The minimum observation time was 4 months and maximum of 22 months. The mean BMI was 26,9 (SD = 4.6). 48% were diagnosed with N-ERD, 83% of patients had comorbid asthma and 72% had any form of allergy. The mean of prior sinus surgeries was 2.9 (SD = 2.0). The mean LMK was 18.5 (SD = 4.4).

**Table 1:** Baseline patient characteristics

Characteristic	Value
No. patients treated with Dupilumab (*n*)	104
Sex: male/female, *n* %	51/53 (49/51)
Age	
Mean (SD)	50.10 (13.64)
Median (IQR)	52 (41.00, 58.00)
Range	14.00, 84
BMI	
Mean (SD)	26.95 (4.65)
Median (IQR)	26 (23.58, 29.72)
Range	18.40, 42
LMK	
*N* (% not missing)	96 (92.31)
Mean (SD)	18.51 (4.49)
Median (IQR)	19 (15.00, 22.00)
Range	7.00, 24
Allergies	75/104 (72%)
Asthma	86/104 (83%)
N-ERD	49/104 (47%)
No. previous sinus surgeries	
Mean (SD)	2.97 (2.01)
Median (IQR)	3 (1.75, 4.00)
Range	0.00, 11
SNOT-22	
Mean (SD)	60.42 (19.36)
Median (IQR)	62 (47.00, 76.25)
Range	5.00, 101
Nasal polyp score (NPS)	
Mean (SD)	4.72 (1.60)
Median (IQR)	4 (4.00, 6.00)
Range	2.00, 8
VAS nasal blockage	
*N* (% not missing)	96 (92.31)
Mean (SD)	6.14 (2.66)
Median (IQR)	6 (5.00, 8.00)
Range	0.00, 10
VAS rhinorrhea	
*N* (% not missing)	96 (92.31)
Mean (SD)	6.51 (2.77)
Median (IQR)	7 (5.00, 9.00)
Range	0.00, 10
VAS facial pain	
*N* (% not missing)	96 (92.31)
Mean (SD)	4.45 (2.92)
Median (IQR)	4 (2.00, 7.00)
Range	0.00, 10
Olfactometry	
Mean (SD)	3.22 (3.65)
Median (IQR)	2 (0.00, 5.00)
Range	0.00, 12
FEV-1	
*N* (% not missing)	100 (96.15)
Mean (SD)	77.71 (14.49)
Median (IQR)	79 (68.75, 88.25)
Range	31.00, 103
ACT	
*N* (% not missing)	67 (64.42)
Mean (SD)	17.58 (5.61)
Median (IQR)	17 (14.50, 22.00)
Range	5.00, 25

### NPS score

**Fig. 1:**
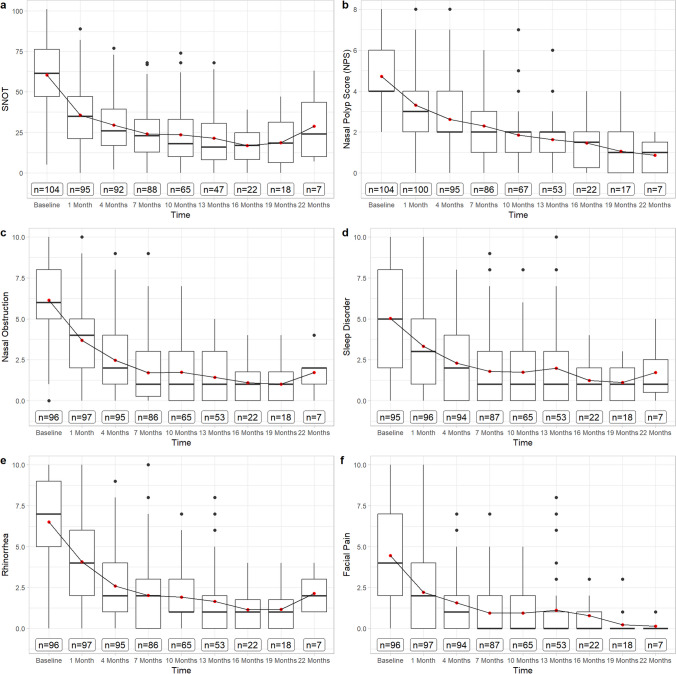
Boxplots displaying the results of primary endpoints **A** SNOT-22, **B** NPS, VAS-scores, **C** nasal obstruction, **D** sleep disorder, **E** rhinorrhea, **F** facial pain in points from baseline to 22 months of Dupilumab therapy. The red dot displays the mean-value, while the black bar displays median and the box the interquartile range

**Fig. 2:**
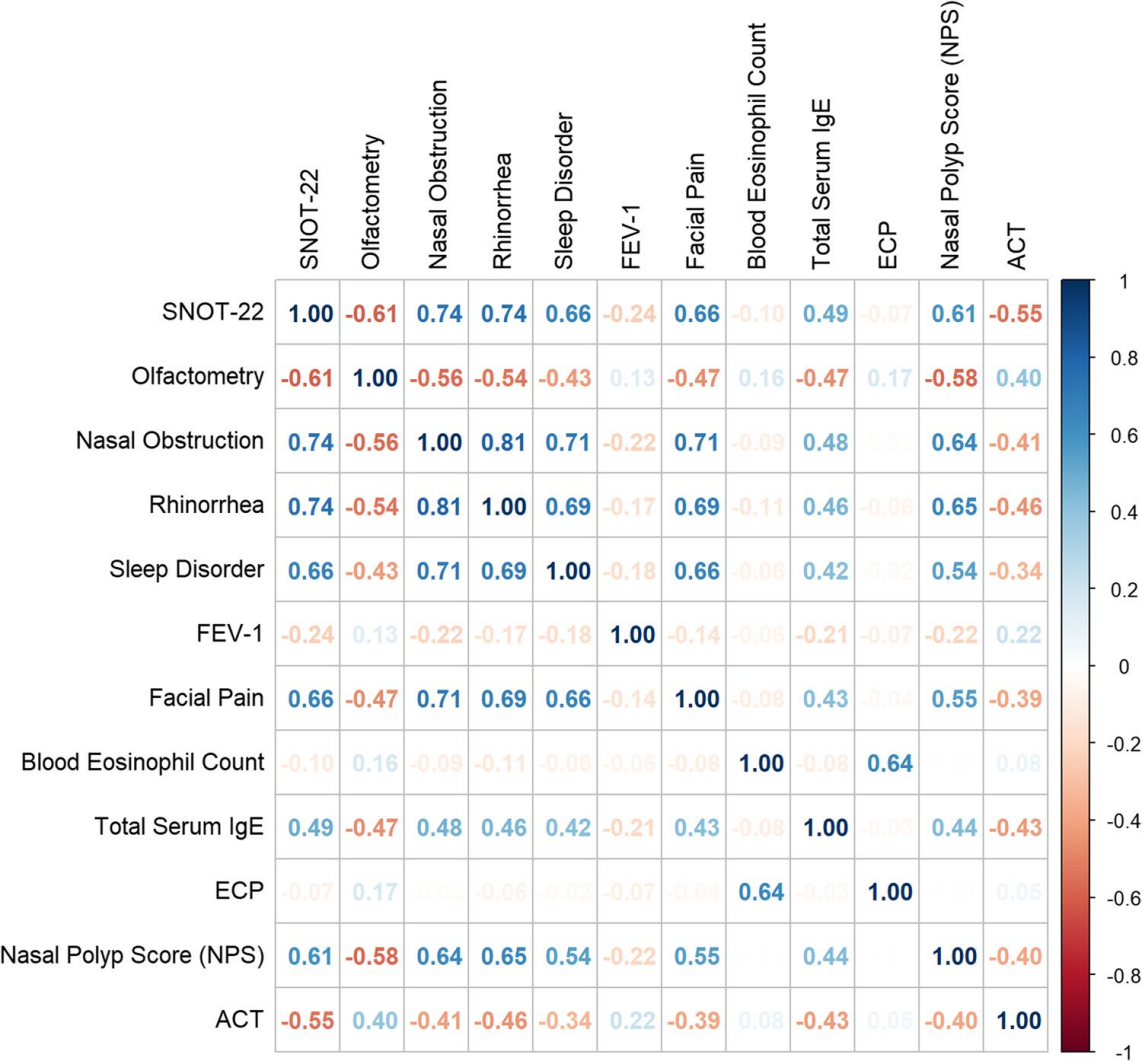
Correlation matrix plot of variables obtained. The color intensities are proportional to the Pearson’s correlation coefficients. 95% confidence intervals for each correlation is displayed in Appendix (Table 3)

At baseline, 85.57% of patients had a NPS of ≥ 4. The NPS decreased from 4.72 (SD = 1.6) to 3.31 (SD = 1.6) after month 1 and to 2.62 (SD = 1.6) at month 4. It decreased further to 1.6 (SD = 1.2) after month 13 and to 0.86 (SD = 0.90) at month 22 (Fig. 1). NPS showed a strong correlation with symptoms of nasal blockage, *r* = 0.64; 95% CI [0.59; 0.69], rhinorrhea, *r* = 0.65; 95% CI [0.59; 0.69] and SNOT-22 scores, *r* = 0.6; 95% CI [0.54; 0.65] (Fig. 2).

### SNOT 22

The initial SNOT-22 score was 60.42 points (SD ± 19.36) before treatment and improved from 35.68 (SD ± 19.34) after 1 month to 29.43 (SD ± 17.77) at month 4 and 21.49 (SD ± 17.67) at month 13. After further decreasing to 16.77 (SD ± 10.75) at month 16, it rose to the level of month 4 with 28.71 (SD ± 22.87) at month 22 (Fig. 1). SNOT-22 showed a strong correlation to the VAS-Scores of nasal blockage, *r* = 0.73; 95% CI [0.69; 0.77], rhinorrhea, *r* = 0.74; 95% CI [0.7; 0.77], facial pain, *r* = 0.66; 95% CI [0.61; 0.71] and sleep disorder, *r* = 0.66; 95% CI [0.61; 0.7] (Fig. 2).

### VAS-scores

All VAS-scores improved remarkably throughout the course of therapy (Fig. 1). Nasal congestion decreased from a baseline of 6.14 points (SD ± 2.66) to 3.68 (SD ± 2.27) after month 1, 1.42 (SD ± 1.55) at month 13 and 1.00 (SD ± 1.08) at month 19 of observation.

Rhinorrhea symptoms improved from 6.51 (SD ± 2.77) before therapy to 1.64 (SD ± 1.88) after month 13 and 1.17 (SD ± 1.34) at month 19, followed by subsequent increase to 2.14 (SD ± 1.35) after month 22.

Facial pain resolved almost completely with a score of 0.14 (SD ± 0.38) after month 22, initially started from 4.45 (SD ± 2.92) before first dosage.

Sleep disorder assessed with 5.03 points (SD ± 3.28) at baseline, decreased constantly to 1.11 (SD ± 1.18) after month 19, followed by subsequent increase to 1.71 points (SD ± 1.80) at month 22.

### Olfactometry, ACT and FEV-1

**Fig. 3:**
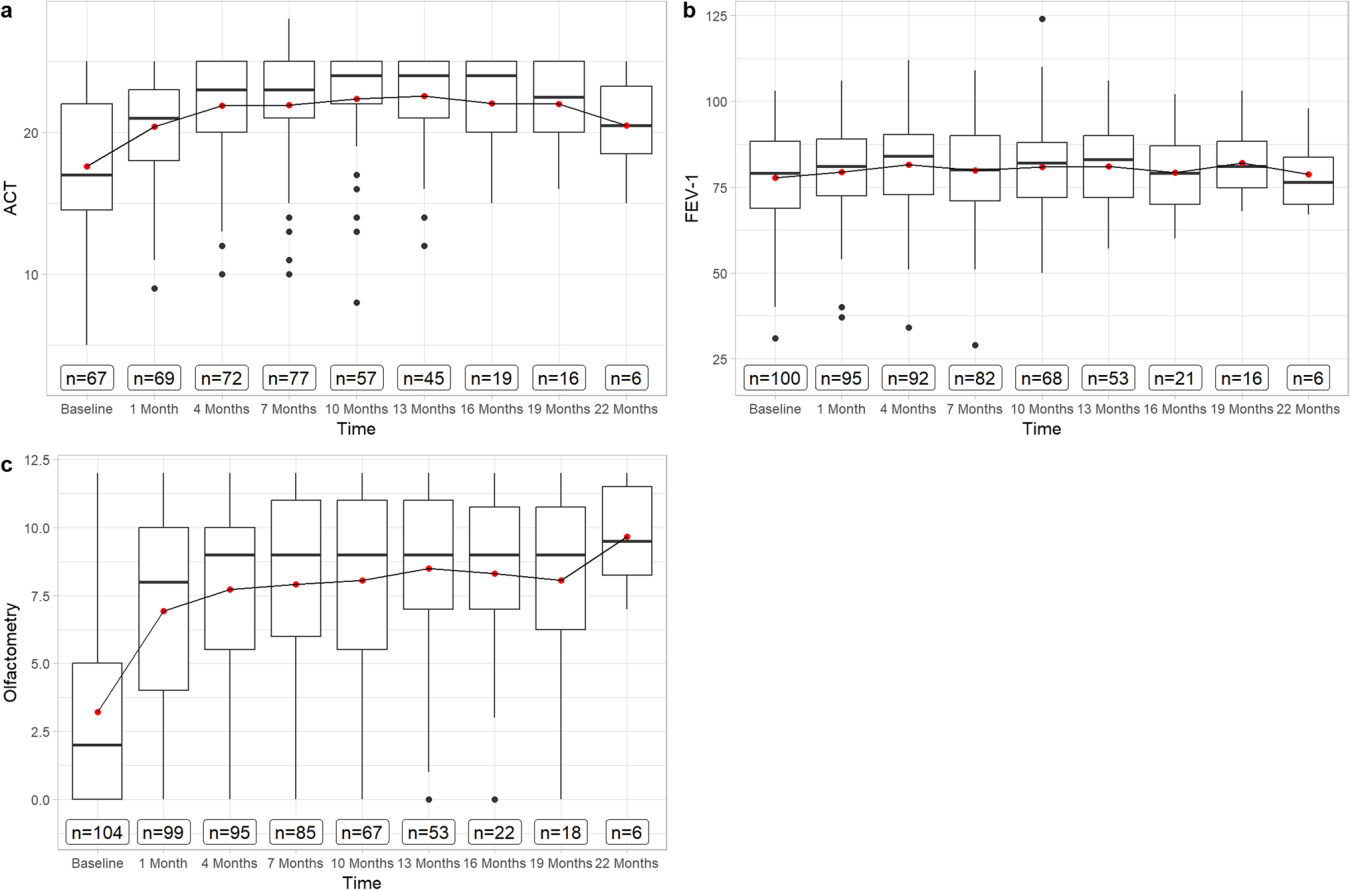
Boxplots displaying results of **A** ACT and **C** olfactometry in points and **B** FEV-1 in per cent over 22 months of Dupilumab treatment. The red dot displays the mean-value, while the black bar displays median-value and the box the interquartile range

Olfaction improved from anosmia to nearly normosmia after a period of 22 months (Fig. 3). At baseline, 75.95% of patients had anosmia (≤ 5 points). The olfactometry score was 3.22 (SD ± 3.65) at baseline, 6.93 points (SD ± 4.01) after month 1 and 8.49 (SD ± 2.87) at month 13. It increased to 9.67 (SD ± 2.07) at month 22. Olfactometry progression under therapy showed a negative correlation with SNOT-22, *r* = − 0,6; 95% CI [− 0.65; − 0.55] and NPS, *r* = − 0.58; 95% CI [− 0.62; − 0.51]. Nasal blockage, rhinorrhea, facial pain, and sleep disorder were found to be inversely correlated with olfactometry results (Fig. 2).

ACT-score improved from 17.58 (SD ± 5.61) to 22.58 (SD ± 3.35) at month 13 and remained stable at 22.00 (SD ± 3.06) at month 19, before decreasing to 20.5 (SD ± 3.71) at month 22 (Fig. 3). ACT-score was correlated with SNOT-22, *r* = − 0.55; 95% CI [− 0.61; − 0.47]. No correlation could be found between ACT-score and FEV-1 (Fig. 2). FEV-1 improved throughout the observation period with a baseline value of 77.71 (SD ± 14.49), slight increase to 82.12 (SD ± 10.29) after month 19, and subsequently decreased to 78.67 (SD ± 11.72) after month 22 (Fig. 3). FEV-1 progression was not correlated to any other variables (Fig. 2).

### Biomarkers ECP, IgE and eosinophils

**Fig. 4:**
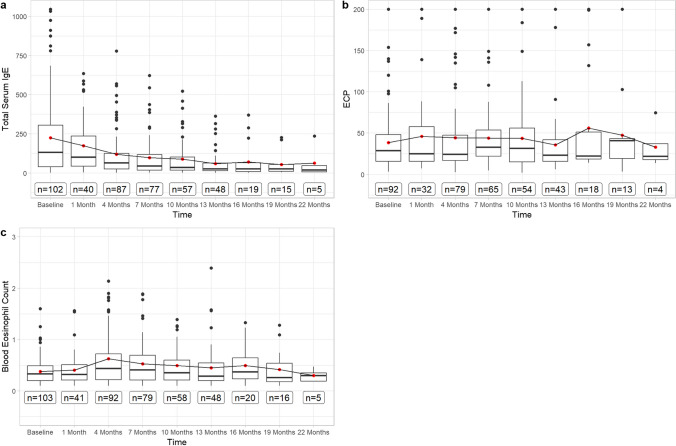
Boxplots displaying the course of Biomarkers **A** total serum IgE (IU/mL), **B** ECP (µg/L) and **C** blood eosinophil count (billion/L) from baseline to 22 months of Dupilumab therapy. The red dot displays the mean-value, while the black bar displays median and the box the interquartile range

**Fig. 5:**
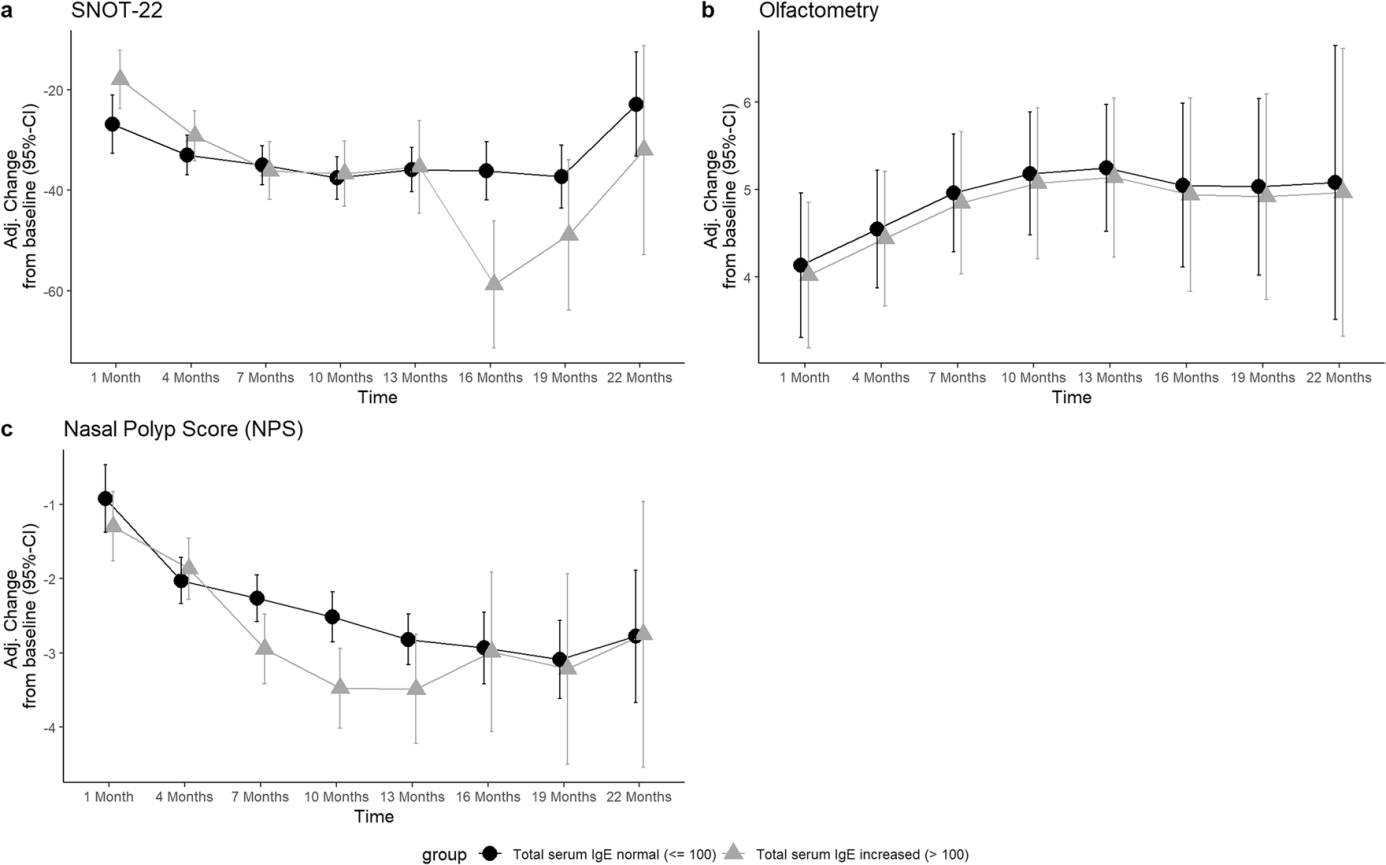
Line plots displaying the adj. changes from baseline of **A** SNOT-22, **B** NPS, **C** ACT in points and **D** FEV-1 in per cent from baseline to 22 months of Dupilumab therapy. The black line displays the course of patient group with normal blood eosinophil count at baseline (< 0.5 billion/L), the grey line shows the course of the group with elevated blood eosinophil count at baseline (> 0.5 billion/L)

Total serum IgE levels decreased progressively from 264.35 IU/mL (SD ± 395.14) to 173.25 IU/mL (SD ± 182.48) after month 1 (Fig. 4), further to 59.43 IU/mL (SD ± 83.39) after month 13 and to 62.92 IU/mL (SD ± 98.12) after month 22. IgE levels did correlate positive with SNOT-22, VAS-Scores, olfactometry and NPS-scores (Fig. 2). Looking at the development of NPS in the two groups with and without increased baseline IgE levels, different courses are noticeable: the patients with initially elevated IgE levels (> 100 IU/mL) had a stronger improvement of NPS after 10 months (− 0.96 points, *p* = 0.001). However, this effect disappeared with further observation to month 22 (Fig. 5).

**Fig. 6:**
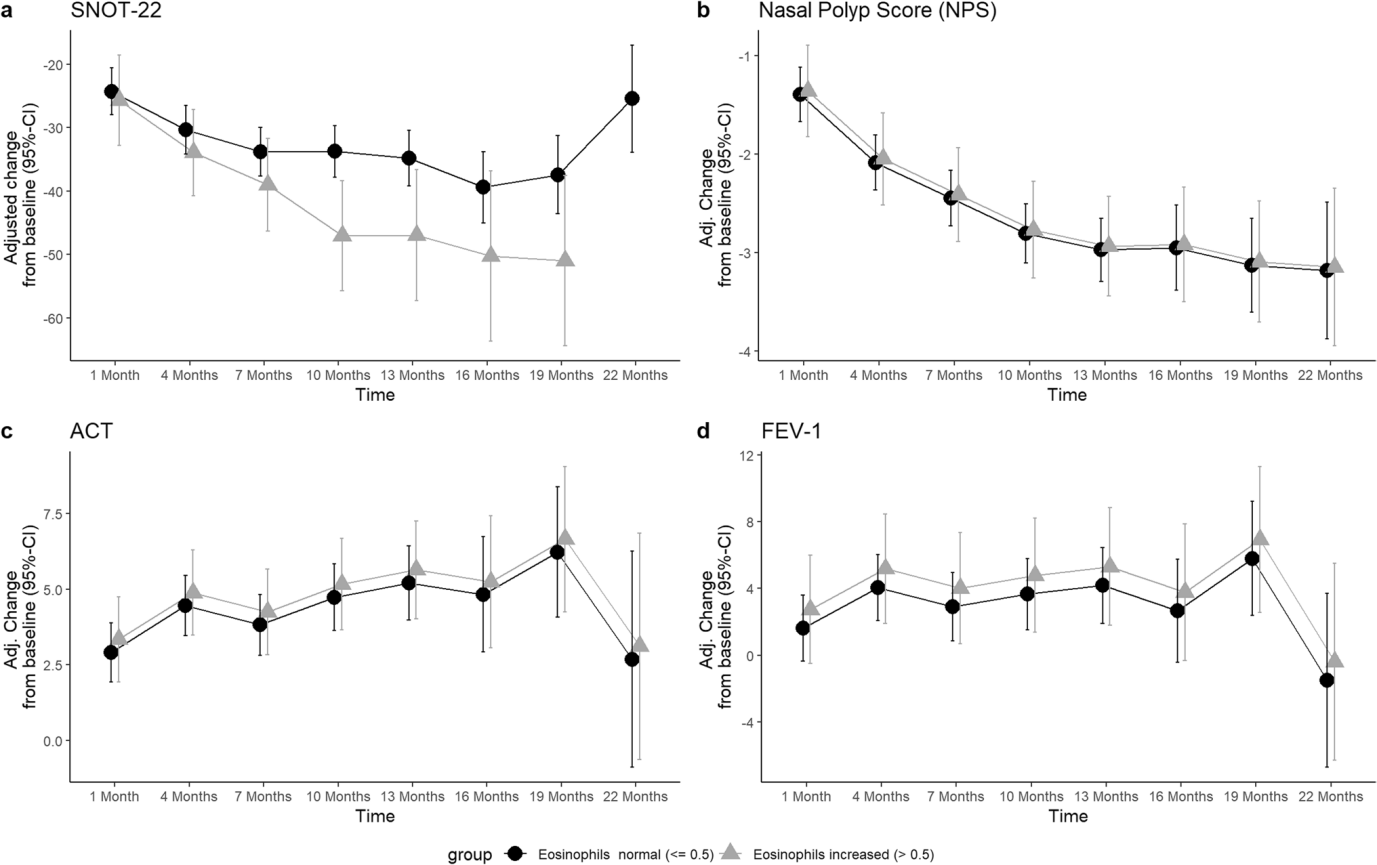
Line plots displaying the adj. changes from baseline of **A** SNOT-22, **B** olfactometry and **C** NPS in points from baseline to month 22 of Dupilumab therapy. The black line displays the course of patient group with normal total serum IgE levels at baseline (< 100 IU/mL), the grey line shows the course of the group with serum IgE levels at baseline (> 100 IU/mL)

**Fig. 7:**
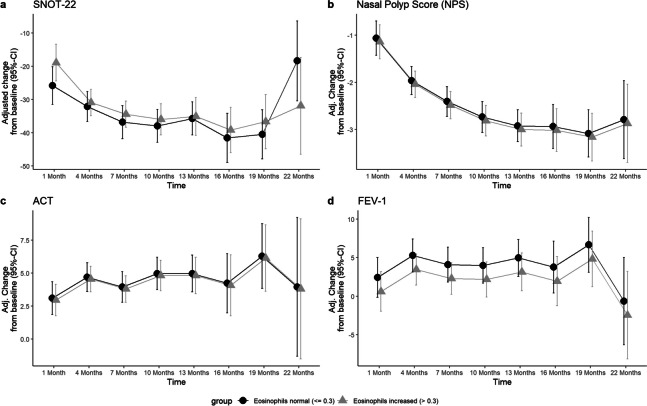
Line plots displaying the adj. changes from baseline of **A** SNOT-22, **B** NPS, **C** ACT in points and **D** FEV-1 in per cent from baseline to 22 months of Dupilumab therapy. The black line displays the course of patient group with normal blood eosinophil count at baseline (< 0.3 billion/L), the grey line shows the course of the group with elevated blood eosinophil count at baseline (> 0.3 billion/L)

BECs in whole blood were 0.38 billion/L (SD ± 0.26) at baseline. Cells counts peaking at month 4 with 0.63 billions/L (SD ± 0.67), thereafter constantly decreasing to 0.45 billions/L (SD ± 0.44) at month 13 and 0.30 billions/L (SD ± 0.12) after month 22 (Fig. 4). No other correlations than to ECP cell count progression was found, *r* = 0.64; 95% CI [0.58; 0.69]. A trend was seen where patients with BECs of > 0.5 billion/L at baseline had a better progression of the SNOT-22 score after 10 months (− 13.28 points difference, *p* = 0.007), 1 year (− 12.19 points, *p* = 0.03) and month 19 (− 13.55 points, *p* = 0.07) (Fig. 6, Table 2). When the threshold was set at 0.3 billion/L, this trend was not observed (Fig. 7). Otherwise, no relevant difference was seen in development of NPS, olfactometry, FEV-1 or ACT between patients with initially elevated BECs (> 0.5 billion/L and > 0.3 billion/L) or physiological cell count during therapy (Figs. 6, 7). Average serum ECP was elevated at baseline with 38.41 µg/L (SD ± 34.91) (Fig. 4). There were no substantial changes in ECP levels throughout observation, neither any correlation to any other variable than BECs or differences in the groups with or without elevated ECP at baseline.

**Table 2:** Upper table displaying the adjusted change from baseline of SNOT-22 score of the groups with increased (> 0.5 billion/L) and normal (< 0.5 billion/L) baseline eosinophil counts over time

Eosinophils increased (> 0.5 billion/L) vs. eosinophils normal (≤ 0.5 billion/L)					
Outcome: SNOT-22 adj. change from baseline					
Time	SNOT-22 adj. change	std. error	lower.95	upper.95	Group
1 month	− 24.26	1.90	− 27.99	− 20.53	Eosinophils normal (≤ 0.5)
1 month	− 25.66	3.62	− 32.76	− 18.57	Eosinophils increased (> 0.5)
4 months	− 30.29	1.95	− 34.12	− 26.47	Eosinophils normal (≤ 0.5)
4 months	− 33.92	3.48	− 40.74	− 27.09	Eosinophils increased (> 0.5)
7 months	− 33.81	1.95	− 37.62	− 29.99	Eosinophils normal (≤ 0.5)
7 months	− 38.99	3.73	− 46.30	− 31.68	Eosinophils increased (> 0.5)
10 months	− 33.74	2.07	− 37.81	− 29.68	Eosinophils normal (≤ 0.5)
10 months	− 47.02	4.43	− 55.70	− 38.35	Eosinophils increased (> 0.5)
13 months	− 34.77	2.24	− 39.16	− 30.38	Eosinophils normal (≤ 0.5)
13 months	− 46.96	5.26	− 57.27	− 36.65	Eosinophils increased (> 0.5)
16 months	− 39.40	2.88	− 45.04	− 33.75	Eosinophils normal (≤ 0.5)
16 months	− 50.23	6.87	− 63.69	− 36.77	Eosinophils increased (> 0.5)
19 months	− 37.43	3.15	− 43.61	− 31.26	Eosinophils normal (≤ 0.5)
19 months	− 50.98	6.85	− 64.41	− 37.55	Eosinophils increased (> 0.5)
22 months	− 25.41	4.31	− 33.85	− 16.97	Eosinophils normal (≤ 0.5)
22 months	NA	NA	NA	NA	Eosinophils increased (> 0.5)

Difference: eosinophils increased (> 0.5 billion/L) vs. normal (≤ 0.5 billion/L)					
Time	Difference	*p* value	95% CI		
1 month	− 1.41	0.7327	(− 9.51, 6.70)		
4 months	− 3.62	0.3683	(− 11.55, 4.30)		
7 months	− 5.18	0.2227	(− 13.53, 3.17)		
10 months	− 13.28	0.0072	(− 22.94, − 3.62)		
13 months	− 12.19	0.0343	(− 23.48, − 0.91)		
16 months	− 10.83	0.1471	(− 25.50, 3.83)		
19 months	− 13.55	0.0736	(− 28.40, 1.30)		
22 months	NA	NA	(NA, NA)		

The lower table displays the difference of adj. change from baseline (95% CI) between the two groups for each time point

### Safety aspects and rescue therapy

**Fig. 8:**
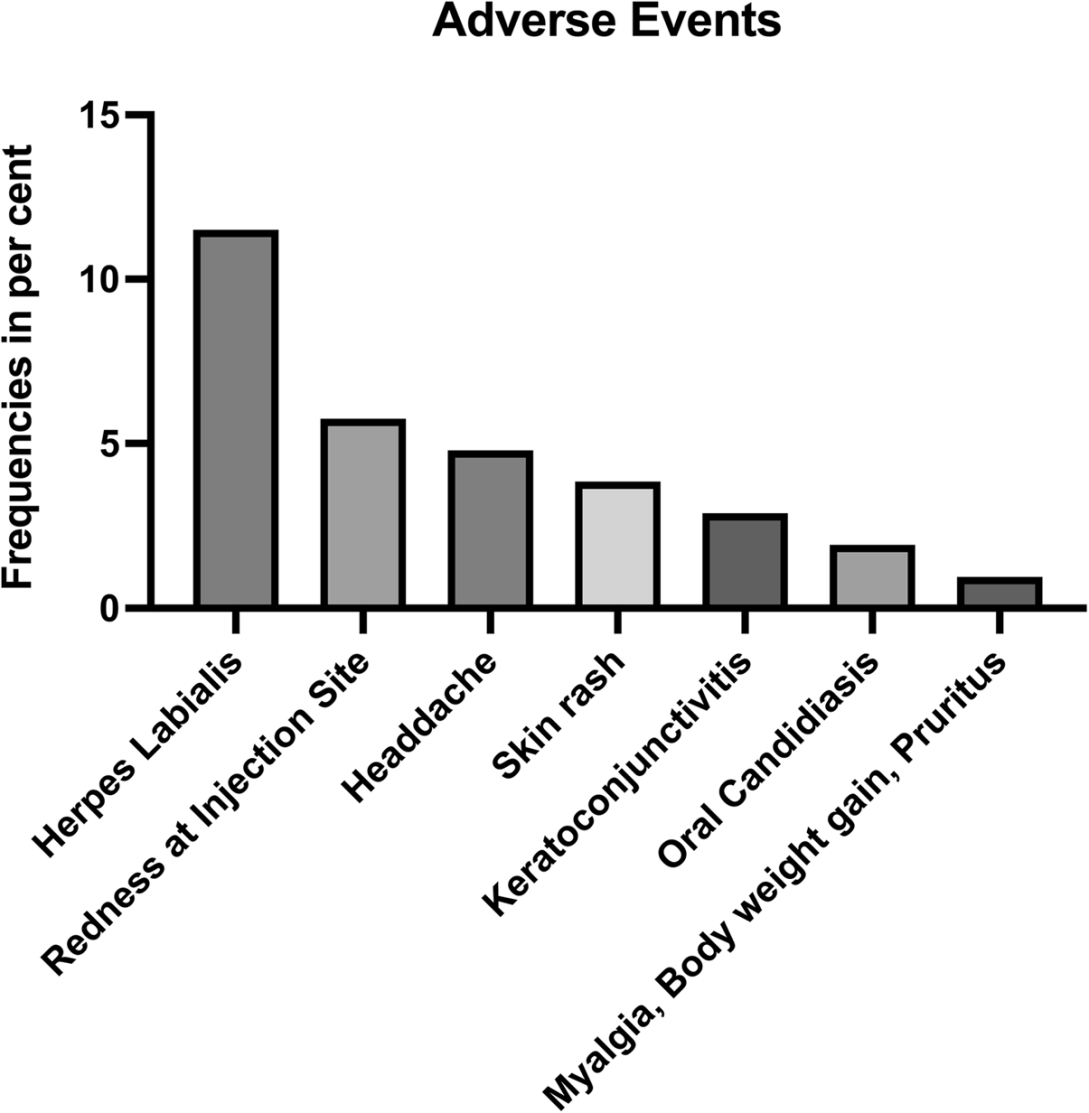
Bar graph of adverse events. The *y*-axis shows the frequencies in percent, while the *x*-axis names the adverse event

Overall, the administration of Dupilumab therapy was well tolerated and no severe treatment events were observed. All observed adverse effects were transient and did not result in discontinuation of therapy. The most commonly reported side effect was a mild herpes labialis in 11.5% of patients, although it remained unclear whether this was directly associated to the therapy (Fig. 8). 5.76% reported redness at the injection site. Brief headache was reported in 4.8% of patients. 3.84% reported diffuse skin rash. Two patients (1.92%) presented with signs of oral candidiasis. Additionally, keratoconjunctivitis was reported by 2.88% of patients, while myalgia, bodyweight gain and pruritus were each reported by one patient (0.96%).

In three cases (2.88%), therapy was discontinued due to non-response, two of these underwent subsequent salvage operation. One patient discontinued therapy at his own request with subjective low response, although VAS-scores as well as blood values showed a positive trend. After 3 months of discontinuation, noticeable worsening of symptoms was observed in this patient (NPS: 1 to 3 points; FEV-1: 75% to 73%; SNOT-22: 59 to 64 points, VAS nasal blockage: 0 to 7 points, rhinorrhea 5 to 7). Additionally, Dupilumab therapy was interrupted in one patient due to unclear interaction with highly needed chemotherapy.

Furthermore, two patients, one with comorbid N-ERD, developed hypereosinophilia (> 3 billion/L) due to treatment without any symptoms or signs of organ damage. These patients were seen for control after 1 month, contrary to the usual observation interval of 3 months. Since a decrease in eosinophils was already observed again, no further therapeutic consequence was drawn.

## Discussion

Since its release, Dupilumab has extended available treatment options for patients with severe uncontrolled CRSwNP. Although there are a few real-world observational studies with 12-month follow-up, there is a need to evaluate the efficacy and safety of Dupilumab beyond 1 year. Long-term observation will reveal whether the effects may diminish over time, whether permanent remission can be achieved or whether side effects may occur. As these reports are limited to date, this study reports long-term follow-up incorporating a larger population and focusing on the analysis of easily obtainable biomarkers in clinical practice settings [4, 24, 25].

Our findings confirm that treatment with Dupilumab results in rapid and consistent improvement throughout the treatment period for up to 22 months. This improvement is evident in the results of the primary endpoint variables SNOT-22 and VAS-scores, as subjective patient outcome measures, as well as in objective endoscopic NPS and olfactometry performance. It is noteworthy that a significant part of therapeutic efficacy manifests within the first 4 months, after which there is only a minor but continuous improvement up to 22 months, suggesting that continuous application of Dupilumab is effective for a longer treatment period. Especially the NPS and olfactometry appear to benefit particularly from prolonged therapy, reaching physiological levels not before month 22 in our observation (0.86 SD ± 0.90 and 9.67 SD ± 2.07). Side effects are only minor in the second year of therapy, and there are no side effects that have not already occurred after initiation of Dupilumab.

These findings increase the long-term understanding of efficacy and safety of Dupilumab therapy, making it possible to provide patients with information about expected effects and when to expect remission, which is important for expectation management and compliance. To further investigate in long-term observation of Dupilumab treatment in patients with severe CRSwNP, a national registry should be established, so that data from the outpatient setting can be included, as more patients can now be started on Dupilumab on an outpatient basis and fewer patients are available for long-term observation in centers. This is of particular interest because treatment with Dupilumab is currently cost-intensive, also considering a possibility of lifelong therapy, and the long-term recurrence of nasal polyps after achieving complete remission and subsequent discontinuation of treatment is still uncertain. With a NPS of 0 in combination with good SNOT and VAS-scores in 19 of our patients at their last documented observation, this might be the appropriate time to consider such an approach. A Japanese study with a small number of patients treated with Dupilumab for atopic dermatitis was able to show that more than half of the observed population achieved long-term remission after an attempted discontinuation of Dupilumab treatment [26]. However, despite both diseases sharing the same pathophysiological basis, drawing conclusions to patients affected by CRSwNP might be incorrect. In contrast, the pivotal study suggests a need for continued suppression of type 2 inflammation to achieve long-term disease control [19]. In our study, however, there was one patient who experienced significant subjective and objective symptom progression 3 months after discontinuation of Dupilumab, supporting the suggestion of continuous suppression.

Impairment of sense of smell is one of the most unpleasant and persistent symptoms in patients with CRSwNP [27]. Studies revealed that loss of olfaction is associated with disease severity and significantly impacts quality of life [28]. Our data align with these findings: SNOT-22- and VAS-scores of nasal blockage and rhinorrhea, as an expression of quality of life, exhibit an inverse correlation with the progression of olfactometry results (Fig. 2). With consistent improvement of olfactometry and NPS, along with a positive correlation, these data suggest a relationship between their development, whereas other studies have linked the change in olfactometry more to tissue eosinophilia-related neurotoxic effects rather than mechanical obstruction [25]. Unfortunately, we were unable to investigate a correlation between tissue eosinophilia and olfactometry as tissue samples have not been collected for each patient and especially not at each timepoint of subsequent follow-up.

Dupilumab has demonstrated its efficacy in addressing several type 2 inflammatory diseases affecting the upper and lower airways at the same time [29]. In CRSwNP patients with comorbid asthma, Dupilumab has shown significant improvements in lung function and asthma control [29]. Consistent with those results, our study demonstrated mild enhancement in FEV-1 and ACT-values, which were found to be independent from baseline BECs.

Biomarkers have a crucial role in guiding therapeutic decisions and monitoring treatment response. However, biomarkers that serve as predictors for the response of Dupilumab therapy in CRSwNP are not existing to date [21]. In this study however, we found that patients with pretherapeutic increased BECs with threshold value at 0.5 billion/L, but not 0.3 billion/L, had a trend of better progression of SNOT-22 score, with a difference of − 13.55 points after 19 months of therapy, which surpasses the 12-point threshold for a minimally clinically important difference (MCID) that was found for the SNOT-22 test in medically managed patients with chronic rhinosinusitis [30]. The potential role of blood eosinophils and their initial cell count as a predictor of therapy response has been discussed. Some studies found better outcomes in NPS and nasal congestion score with elevated baseline cell counts treated with Mepolizumab and Benralizumab, though without statistical significance [31–33], while others attested no further capability in identifying responsiveness [19, 22]. Bertlich et al. found the initial BECs to have at least some influence on the development of variables, although no underlying systemic effect was assumed [34], whereas others found higher baseline BECs to predict better outcomes in SNOT-22 scores [35, 36]. Along with these, our findings provide further evidence that baseline BECs > 0.5 billion/L may be a suitable biomarker for predicting, at least subjective, responsiveness to Dupilumab in patients with severe CRSwNP. Nonetheless, the result must be seen in the context that only SNOT-22 and none of the other variables showed this result (Fig. 5). Temporary elevations of BECs in patients undergoing Dupilumab treatment have been previously reported in the SINUS trials and were observed to resolve spontaneously within a few months without causing significant symptoms [19, 37, 38]. However, there have been reported cases of hypereosinophilic syndrome (HES) or eosinophilic granulomatosis with polyangiitis (EGPA) in some patients [38]. Consistent with those observations, we saw a transient increase in cell counts after initiation of therapy, which subsequently regressed back to baseline levels during the course. A transient hypereosinophilia with a cell count exceeding 3 billion/L was observed in two patients. However, this condition was self-limiting and resolved within a period of 3 months, without causing any noticeable symptoms. Regardless of hypereosinophilia, both subjective and objective outcomes improved consistently. Studies investigating the management of this condition suggest that hypereosinophilia can be considered benign when the BEC is less than 3 billion/L and no involvement of organs is seen. However, when the cell counts exceeded 3 billion/L, short-term corticosteroid therapy is recommended to reduce the number of eosinophils in the blood and prevent potential organ involvement [39]. The transient increase of eosinophil cell count can be attributed to the hypothesis of Dupilumab blocking eotaxin-3, a chemokine which specifically attracts eosinophils to the site of inflammation [40]. Consequently, more eosinophil cells remain in peripheral blood. Nevertheless, the lack of response in BECs after initiating therapy, while at the same time significant improvements in NPS, SNOT-22, and VAS-scores were seen, indicates a discrepancy between BECs and its influence on disease severity. These findings suggest one the one hand, that BECs are not reflecting those clinical improvements and effective suppression of type 2 inflammation processes, and on the other, that suppressing BECs may not be required to achieve therapeutic success. Either way, their ability to be used as a biomarker for monitoring therapeutic success can be denied.

Consistent with previous trials showing a reduction in serum IgE levels in patients treated with Dupilumab for CRSwNP, total serum IgE levels decreased continuously until the end of the observation period in our study [11, 19, 25]. Thereby, the progression of total serum IgE levels showed moderate correlations with all clinical outcome parameters, with exception of FEV-1 and BECs (Fig. 2). While improvements in clinical symptoms are paramount, these findings suggest that insights into the improvement of the symptoms and inflammation severity could be inferred through the biomarker IgE, making it a good follow-up parameter for therapy response. On the other hand, the study showed that the pretherapeutic IgE levels do not allow for any conclusions regarding the long-term course of subsequent therapy, although a better NPS response in the first year was found in patients with elevated IgE levels. Taking into account the better SNOT-22 response in patients with elevated BECs, one may conclude that the more pronounced the type 2 endotype, the better the treatment response in the first year.

ECP is a cytotoxic secretory protein with bactericidal and antiviral properties, produced and released by activated degranulating eosinophils and considered as meaningful marker of eosinophilic inflammation [41]. The connection between ECP and activated eosinophils in the blood is also evident in our study, *r* = 0.64; 95% CI [0.58, 0.69] [42]. Higher levels of ECP have been reported in recurrent CRSwNP, potentially contributing to epithelial damage in nasal mucosa [42]. Pre-interventional ECP levels were found to be a reliable prognostic indicator of polyp recurrence after surgery [43]. Whether it can also predict the response to Dupilumab therapy has, to the best of our knowledge, not yet been investigated. In our study, we observed that the progression of the clinical variables was irrespective of the initial ECP levels. There were no clinically relevant changes in serum ECP levels besides a small transient increase. Mean ECP levels remained elevated from baseline throughout the entire observation period. No correlations were found to the clinical parameters. In conclusion, these observations suggest that serum ECP cannot be used to predict or monitor treatment response. Instead, the ECP levels in nasal secretions may serve as a more suitable biomarker to monitor the success of Dupilumab therapy, since it was shown to decrease under therapy before [11, 19]. For this purpose, further investigations with the collection of nasal secretion samples and statistical correlations are needed.

Since BECs and serum ECP did not provide any indication useful for monitoring the success of therapy, the question arises whether routine blood sampling at each follow-up could also be dispensed with. Especially in the field of outpatient care of CRSwNP patients treated with Dupilumab, the reduction of laboratory controls could bring significant relief and cost reduction. In general, Dupilumab had only very few side effects, and even in the very rare case of hypereosinophilia, patients did not only remain asymptomatic but consistently improved their clinical condition. Unfortunately, predicting possible hypereosinophilia is still challenging. Wechsler et al. and Ryser et al. proposed that only patients with higher baseline BECs may be at greater risk of developing transient hypereosinophilia [35, 44]. Rampi et al. found the 2-months value of BECs as a possible predictor of long-lasting hypereosinophilia in patients treated with Dupilumab [45]. Their results showed that hypereosinophilia did not develop within 2 years if the eosinophil count did not exceed 1.5 billion/L after 2 months. Incorporating our observation, one may suggest that laboratory controls of biomarkers may only be necessary for the first months of therapy, if normal values are observed during this period. Thereafter, a targeted anamnesis for potential eosinophil-related morbidity might provide similar safety as a laboratory check. If maintaining routine blood tests to monitor therapy success, serum IgE emerges as a more valuable parameter compared to the other biomarkers.

Several potential limitations apply to our study. The data for this study was collected retrospectively which resulted in missing data that could potentially impact the validity of our analyses. Furthermore, we were unable to control if all patients adhered to the prescribed administration of Dupilumab and intranasal corticosteroids during the treatment period. Finally, one major limitation should be pointed out: towards the end of observation period, only very few patients were available for observation.. This reduction in observations caused the data, like the subjective outcome variables SNOT-22-Score and VAS-scores, to inexplicably deviate towards poorer values at month 22. This deviation may potentially be explained by the fact that only the most severely affected patients are seen for follow-ups at our specialized center for an extended period, rather than receiving outpatient treatment. Their values may deviate towards the worse and may lead to a selection bias. The reduced sample size primarily results from the majority of patients being referred to outpatient care after only 1 year, as outpatient monitoring and initiation of Dupilumab is increasingly possible, often meaning shorter travel distances for patients. The strength of this study is the ability to demonstrate the effect of Dupilumab on CRSwNP patients with a larger and diverse population and a long-term observation period. This applies in particular for the analysis of the collected blood parameters in connection with clinical parameters, as they can be easily obtained in everyday settings.

## Conclusion

Our long-term, real-world cohort study consistently supports Dupilumab as an effective add-on treatment option for severe, uncontrolled CRSwNP, showing persistently positive subjective and objective outcomes. Therapeutic efficacy is mainly established within the first 6 months of treatment and continues up to the end of this study at 22 months. No new or serious side effects occurred during the second year of observation. Predicting therapy outcome based on biomarkers remains challenging. While increased pretherapeutic blood eosinophil counts may predict particularly good development of SNOT-22 values, pretherapeutic total serum IgE levels and ECP do not have any long-term prediction capacity. While total serum IgE may work as a follow-up parameter for treatment response, BECs and ECP are not suitable for monitoring the therapy success. The necessity of routine blood sampling for biomarker control may be discussable after the first quarter year of therapy.

## Data Availability

The authors confirm that the data supporting the findings of this study are available within the article [and/or] its supplementary materials.

## Appendix

See Table 3.

**Table 3:** Table displaying each Pearson correlation coefficient with 95% CI

Parameter 1	Parameter 2	*r* value	95% CI low	95% CI high
ECP	ACT	0.0524580	− 0.0580357	0.1616820
ECP	NPS	− 0.0098143	− 0.1080082	0.0885692
Eosinophil count	ACT	0.0788694	− 0.0236920	0.1797880
Eosinophil count	ECP	0.6417485	0.5802060	0.6960045
Eosinophil count	NPS	− 0.0075448	− 0.0992033	0.0842406
Eosinophil count	Total serum IgE	− 0.0827310	− 0.1738401	0.0097822
FEV1	ACT	0.2171137	0.1220158	0.3082615
FEV1	ECP	− 0.0736920	− 0.1719883	0.0260573
FEV1	Eosinophil count	− 0.0553263	− 0.1470129	0.0373029
FEV1	NPS	− 0.2197115	− 0.2995782	− 0.1367868
FEV1	Total serum IgE	− 0.2093003	− 0.2970716	− 0.1180216
FEV1	VAS facial pain	− 0.1440424	− 0.2276126	− 0.0583658
NPS	ACT	− 0.3989524	− 0.4767187	− 0.3149900
Olfactometry	ACT	0.3998594	0.3160663	0.4774626
Olfactometry	ECP	0.1661676	0.0691205	0.2600991
Olfactometry	Eosinophil count	0.1604117	0.0699112	0.2482931
Olfactometry	FEV1	0.1297257	0.0449720	0.2126253
Olfactometry	NPS	− 0.5761417	− 0.6297088	− 0.5171215
Olfactometry	Total serum IgE	− 0.4667713	− 0.5360055	− 0.3913004
Olfactometry	VAS facial pain	− 0.4667221	− 0.5308513	− 0.3972781
Olfactometry	VAS nasal blockage	− 0.5640527	− 0.6194638	− 0.5030490
Olfactometry	VAS rhinorrhea	− 0.5385614	− 0.5963764	− 0.4751875
Olfactometry	VAS sleep disorder	− 0.4267009	− 0.4941056	− 0.3541970
SNOT-22	ACT	− 0.5493925	− 0.6134440	− 0.4781605
SNOT-22	ECP	− 0.0679372	− 0.1671357	0.0326230
SNOT-22	Eosinophil count	− 0.1043739	− 0.1957404	− 0.0112110
SNOT-22	FEV1	− 0.2408918	− 0.3211581	− 0.1571892
SNOT-22	NPS	0.6055643	0.5485798	0.6569490
SNOT-22	Olfactometry	− 0.6082021	− 0.6593622	− 0.5514354
SNOT-22	Total serum IgE	0.4943740	0.4197797	0.5623345
SNOT-22	VAS facial pain	0.6646510	0.6136885	0.7100983
SNOT-22	VAS nasal blockage	0.7351805	0.6929593	0.7723764
SNOT-22	VAS rhinorrhea	0.7436745	0.7025754	0.7798288
SNOT-22	VAS sleep disorder	0.6628018	0.6115715	0.7084975
Total serum IgE	ACT	− 0.4278115	− 0.5085724	− 0.3395887
Total serum IgE	ECP	− 0.0348335	− 0.1328001	0.0638072
Total serum IgE	NPS	0.4351405	0.3569738	0.5072555
VAS facial pain	ACT	− 0.3926732	− 0.4715864	− 0.3075207
VAS facial pain	ECP	− 0.0370540	− 0.1362376	0.0628650
VAS facial pain	Eosinophil count	− 0.0830929	− 0.1745982	0.0098355
VAS facial pain	NPS	0.5505733	0.4883620	0.6072164
VAS facial pain	Total serum IgE	0.4251980	0.3451603	0.4990960
VAS nasal blockage	ACT	− 0.4133396	− 0.4905498	− 0.3296900
VAS nasal blockage	ECP	− 0.0163186	− 0.1156888	0.0833749
VAS nasal blockage	Eosinophil count	− 0.0869825	− 0.1782926	0.0058128
VAS nasal blockage	FEV1	− 0.2220212	− 0.3026189	− 0.1382706
VAS nasal blockage	NPS	0.6432180	0.5904436	0.6905152
VAS nasal blockage	Total serum IgE	0.4799418	0.4043408	0.5490288
VAS nasal blockage	VAS facial pain	0.7130166	0.6687934	0.7522119
VAS nasal blockage	VAS rhinorrhea	0.8060877	0.7743486	0.8337808
VAS nasal blockage	VAS sleep disorder	0.7086618	0.6638103	0.7484343
VAS rhinorrhea	ACT	− 0.4565836	− 0.5299951	− 0.3763793
VAS rhinorrhea	ECP	− 0.0585439	− 0.1571859	0.0412546
VAS rhinorrhea	Eosinophil count	− 0.1138837	− 0.2044724	− 0.0213609
VAS rhinorrhea	FEV1	− 0.1692348	− 0.2519537	− 0.0840610
VAS rhinorrhea	NPS	0.6516382	0.5998061	0.6980244
VAS rhinorrhea	Total serum IgE	0.4553847	0.3777150	0.5266906
VAS rhinorrhea	VAS facial pain	0.6879684	0.6406949	0.7300455
VAS rhinorrhea	VAS sleep disorder	0.6927166	0.6459191	0.7343275
VAS sleep disorder	ACT	− 0.3352117	− 0.4186711	− 0.2461373
VAS sleep disorder	ECP	− 0.0211095	− 0.1208003	0.0790029
VAS sleep disorder	Eosinophil count	− 0.0626460	− 0.1548051	0.0305939
VAS sleep disorder	FEV1	− 0.1788199	− 0.2613455	− 0.0936990
VAS sleep disorder	NPS	0.5433417	0.4803402	0.6007651
VAS sleep disorder	Total serum IgE	0.4234089	0.3430448	0.4976194
VAS sleep disorder	VAS facial pain	0.6633140	0.6131118	0.7081805
